# Blindingly obvious (once revealed): a novel approach to blinding medicinal products for injection

**DOI:** 10.1186/1745-6215-16-S2-O51

**Published:** 2015-11-16

**Authors:** Julia Sanders, Julia Townson, Nadine Aawar

**Affiliations:** 1 Cardiff University, Cardiff, UK

## Background

Blinding is a pre-requisite to high standard clinical trials of medicinal products (CTIMPs), but this requirement, can on occasions, present complex challenges in placebo matching and delivery. OBS2 is a trial evaluating the effectiveness of early fibrinogen administration in the management of complex postpartum haemorrhage. Fibrinogen concentrate is a white powder that froths distinctively on reconstitution and a suitable placebo, that could match these characteristics, could not be identified during the trial design stage.

## Methods

The OBS2 study team developed an innovative method for the reconstitution and administration of Fibrinogen concentrate that enabled the safe administration of the IMP whilst maintaining blinding without the use of a product similar in appearance in Fibrinogen. The method follows a three stage process of adding dilutant to sleeved ampoules, drawing the reconstituted IMP into black syringes, and excess air expelled into a starch. The novel blinding method ensured that the IMP was administered safely without the clinician viewing the IMP ampoule contents, syringe contents, or reconstituted IMP expelled from the syringe with any excess air.

## Implications for trial design

The method of blinding developed for OBS2 provides an inexpensive method of blinding with potential to be utilised in other CTIMPs where reconstitution of drugs is required but a suitable placebo, either cannot be identified, or only identified at substantial cost. The full novel method of IMP reconstitution will be described and demonstrated using trial training materials including video.

